# Healing Through History: a qualitative evaluation of a social medicine consultation curriculum for internal medicine residents

**DOI:** 10.1186/s12909-021-02505-1

**Published:** 2021-02-08

**Authors:** Joel Bradley, David Styren, Abigail LaPlante, John Howe, Sienna R. Craig, Emily Cohen

**Affiliations:** 1 White River Junction VA Medical Center (10E2E), 215 N Main Street, White River Junction, VT 05009 USA; 2 Geisel School of Medicine at Dartmouth, Hanover, NH USA; 3 Children’s Hospital at Dartmouth-Hitchcock, Lebanon, NH USA; 4 Dartmouth Hitchcock Internal Medicine Residency Program, Lebanon, NH USA; 5 Department of Medicine, The Elliot Hospital, Manchester, NH USA; 6 Tufts University School of Medicine, Boston, MA USA; 7 Dartmouth College Department of Anthropology, Hanover, NH USA; 8 Pulmonary & Critical Care Medicine Fellowship, University of Michigan, Ann Arbor, MI USA

**Keywords:** Social medicine, Social determinants of health, Narrative medicine, Meaning in medicine, Co-production

## Abstract

**Background:**

Social context guides care; stories sustain meaning; neither is routinely prioritized in residency training. Healing Through History (HTH) is a social medicine consultation curriculum integrating social determinants of health narrative into clinical care for medically and socially complex patients. The curriculum is part of an internal medicine (IM) residency outpatient clinical rotation at a Veterans Health Administration hospital. Our aim was to explore how in-depth social medicine consultations may impact resident clinical practice and foster meaning in work.

**Methods:**

From 2017 to 2019, 49 categorical and preliminary residents in their first year of IM training were given two half-day sessions to identify and interview a patient; develop a co-produced social medicine narrative; review it with patient and faculty; and share it in the electronic health record (EHR). Medical anthropologists conducted separate 90-min focus groups of first- and second-year IM residents in 2019, 1–15 months from the experience.

**Results:**

46 (94%) completed HTH consultations, of which 40 (87%) were approved by patients and published in the EHR. 12 (46%) categorical IM residents participated in focus groups; 6 PGY1, and 6 PGY2. Qualitative analysis yielded 3 themes: patient connection, insight, and clinical impact; clinical skill development; and structural barriers to the practice of social medicine.

**Conclusions:**

HTH offers a model for teaching co-production through social and narrative medicine consultation in complex clinical care, while fostering meaning in work. Integration throughout training may further enhance impact.

**Supplementary Information:**

The online version contains supplementary material available at 10.1186/s12909-021-02505-1.

## Background

Social medicine – the practice of deliberate inquiry into the social context of patient care – is challenging to teach, and not routinely emphasized in the clinical learning environment [1–3]. Yet social medicine techniques, including the use of qualitative interviews and the formulation of individualized care plans, provide the raw material for addressing social determinants of health and creating more equitable health care outcomes [4]. This may be particularly true in our most medically and socially complex patients, for whom a multidisciplinary, individualized approach appears to be the most effective way to provide better care at lower cost [5]. The lack of formalized social medicine training signals a gap between curriculum and practice in contemporary health systems, opening an opportunity for study and innovation. At stake are not just patient experience, health, and cost, but also the experience of providing care, which consists of “joy and meaning in work”: the idea that when those working in a health care system find joy, a sense of accomplishment, and belief in the importance of their daily work, they are more engaged and better equipped to collaborate in providing daily patient care [6]. Logically, this notion of meaning in work has emerged as an increasing focus of international health systems dialogues about workforce wellness, engagement and patient care outcomes [1, 2, 6]. Because effective care systems require both wellness and clinical excellence, there has been a growing emphasis on the cultivation of meaning in graduate medical education, as in the Accreditation Council on Graduate Medical Education’s *Back to Bedside* initiative, which reemphasizes the traditional core of medical practice: the healing relationship between clinician and patient [7, 8].

At the center of the relationship between clinician and patient are stories of illness, which illuminate social factors impacting health. Elicitation requires the skills and methods of narrative medicine:

A scientifically competent medicine alone cannot help a patient grapple with the loss of health or find meaning in suffering. Along with scientific ability, physicians need the ability to listen to the narratives of the patient, grasp and honor their meanings, and be moved to act on the patient’s behalf. This is narrative competence…the competence that human beings use to absorb, interpret, and respond to stories [9].

As with all competencies critical to clinical practice, the development of a social medicine approach to clinical care requires experience to achieve such “narrative competence,” but few structured opportunities exist for mentored practice in the clinical learning environment [10].

### Setting and participants

The Healing Through History (HTH) curriculum is part of a required outpatient clinical rotation for Dartmouth-Hitchcock internal medicine (IM) residents at the Veterans Health Administration Hospital in White River Junction, Vermont (WRJVA): a 60-bed hospital integrated with primary care, subspecialty, mental health and surgery clinics serving as a regional hub for 20,000 Veterans living across predominantly rural areas of Northern New England. The Dartmouth-Hitchcock internal medicine residency program is comprised of both “categorical” residents in their first year of training (the first of 3 years to become board-eligible in internal medicine), and “preliminary” residents from psychiatry and anesthesia (who complete a single year of internal medicine before moving into subspecialty training). Between August 2017 and June 2019, 49 first-year residents participated under the guidance of two core faculty mentors: a hospitalist (JB) and a primary care physician (EC).

## Methods

### Program description

HTH is a novel social medicine consultation curriculum integrating social determinants of health into clinical care for medically and socially complex patients. Our aims were first to understand how in-depth social medicine consultations conducted by first-year IM residents for medically and socially complex patients might impact residents’ knowledge, skills, attitudes and subsequent practice of social medicine; and, second, to evaluate whether the narrative process of eliciting, listening, writing and reflecting on social context enhanced connection to complex patients, and fostered meaning in work.

First–year residents are given 2 half-day clinic sessions in which to identify and interview a patient; write a narrative social medicine consult note, reviewed by faculty; and obtain patient approval to include it in the EHR and share it with the clinical team.

The HTH intervention consists of three phases: background reading and patient selection; interviewing, writing, editing, and approval; and reflection. At the outset, we send a standardized e-mail with a brief project description, objectives, and selected articles (Supplementary Appendix 1), and a simplified and evidence-based patient interview guide (Supplementary Appendix 2). Core faculty suggest ways of identifying patients, including direct peer referral from colleagues or attending physicians working in the hospital inpatient units or ICU, or from primary care teams. Residents focus on patients who have been hospitalized 2 or more times in the preceding year, or who are perceived by their clinical team to be at high risk for hospitalization based on chronic disease burden and/or social factors.

After identifying a patient, residents are entrusted with contacting the patient and current clinical care team to gather context, and to determine the proper timing and setting for interview (e.g. in an adequately private hospital room, family meeting space, or outpatient clinic room). Residents first describe the project to the patient and obtain verbal consent; if consent is given, residents conduct the interview. Afterwards, residents craft a consultation note, written in the third person. Once drafted, residents meet with HTH faculty to read and edit the note together, and reflect on the experience. The final consult note is then printed and handed to the patient, or mailed to them, asking them to review, propose edits or amendments, and ultimately, consent to inclusion in the medical record. The resident then makes any requested modifications, and uploads the note into the VA EHR, including the date of the interview. The patient is always offered a copy of the final note: for their records, or to share with family. We use a note title called “My Story,” which in turn is listed in a centralized part of the medical record called “Postings,” allowing the care team to find the note, reference it, and addend it in the future, inviting further co-production (Supplementary Appendix 3).

Mentorship is offered by core faculty throughout the process, as questions arise about patient selection, the interview and writing process, findings, and the final consult note. When reviewing the initial draft of the note with residents, faculty ask critical questions, prompting residents to consider how what they have learned about social context and the narrative of illness might impact clinical care, offering insight and knowledge about the local care delivery system, including resources unknown to residents. Throughout, faculty strive to maintain a non-directive educational stance, affording residents autonomy in how they navigate patient interviews and write up this experience. The only requirement is that residents begin with an open-ended, patient-directed “life story,” proceed to a structured social and military history, and conclude with an assessment and recommendations co-produced with the patient. Some residents complete their interviews in more than one episode due to interruptions in the course of clinical care, scheduling limitations, patient request, or other factors.

HTH was funded by the ACGME Back to Bedside grant program and conducted from 2017 to 2019. A description of the HTH curriculum—predating qualitative analysis—was presented as a poster at the 2019 ACGME Annual Educational Conference. The project was approved by the WRJVA Medical Center Institutional Review Board.

### Program evaluation

We collaborated with the Dartmouth College Department of Anthropology to qualitatively assess the educational outcomes and impact on the residents. Using a modified Delphi approach, anthropologists and core faculty developed a semi-structured focus group guide consisting of five open-ended questions (Supplementary Appendix 4).

We obtained permission from IM residency leadership to offer optional 90-min focus groups to first- and second-year residents. Timing was deliberately coordinated with IM residency schedules to support maximum participation, and reflects convenience sampling. To avoid possible confounding and bias introduced by training program type and variance in practice setting and curriculum, only IM residents were asked to participate in focus groups. Invitations were sent via e-mail to 26 internal medicine residents who had done HTH consultations during their first year, on a rolling basis, 1–15 months prior; a single follow-up reminder was sent. Food was provided during the focus groups, but no further incentive was offered. 2 focus groups were conducted in 2019 by an anthropology research assistant (AL): for second-year residents in February of their second year (8–15 months following the experience), and for first-year residents in April of their first year (0–9 months following the experience). The anthropology research assistant had no connection to the residency program and had not met any of the participants prior to the focus group. No core faculty were present for either focus group to minimize the Hawthorne effect [11] and assure confidentiality of subjects. All residents participated voluntarily and signed consent forms.

Focus groups were audio-recorded and manually transcribed; identifiable information was modified and names were replaced with pseudonyms to protect participant confidentiality (AL). Transcripts were analyzed using conventional content analysis [12]; no coding programs were used. Two authors (AL and JB, a hospitalist physician with knowledge of qualitative methods) annotated them independently and derived key themes from the text. Sub-themes were then independently derived through inductive coding [13, 14], defined and reconciled into 3 principle themes (Supplementary Appendix 5). Codes developed during analysis of the first focus group transcript were applied to the second; authors met to review and validate code application, and resolved discrepancies through open discussion; there were no discrepancies that could not be readily resolved. Representative quotations were selected for each theme to enhance context, and reviewed afterward by all authors for consensus.

## Results

### HTH social medicine consultations

Forty-nine first-year internal medicine and preliminary residents participated in the curriculum during the 2017–2018 and 2018–2019 academic years, constituting 70% of the categorical IM program (not all interns rotate at the VA in their first year). In total, 46 (94%) identified, interviewed, and wrote an HTH consultation on a medically and socially complex Veteran. Only 3 (6%) of 49 residents were unable to arrange and conduct an HTH interview due to truncated rotations. Of the 46 completed interviews, 40 (87%) were approved and published in the EHR; 4 (9%) patients did not respond to a mailed request for editing and approval; and 2 (4%) patients declined to have the note included in their medical record. In follow up discussion with faculty, both patients cited specific disagreements with note recommendations (e.g. patient rejection of suggestion for long-term care). 9 patients (23%) who participated in a resident narrative consultation died within 6 months of their interview.

### Resident focus groups

Forty-six first year residents conducted full HTH consultations from 2017 to 2019; 12 of 26 categorical IM residents (46%) participated in qualitative focus groups. 6 (43%) first-year internal medicine residents who had completed HTH consults in the July 2018–June 2019 academic year participated in a focus group in February 2018, 0–9 months from the experience. 6 (50%) second-year internal medicine residents who had completed HTH consults in the September 2017–June 2018 academic year participated in a focus group in April 2019, 9–15 months from the experience. Analysis of focus group transcripts yielded 3 major themes: patient connection, insight, and clinical impact; clinical skill development; and structural barriers to the practice of social medicine. A complete list of representative quotations supporting each theme are in Table 1.

**Table 1:** HTH Resident Focus Group Themes and Representative Quotations

Theme	Focus group quotations from Dartmouth-Hitchcock Internal Medicine residents First-year (R1, n=6; April 2019) and Second-year (R2, n=6; February 2019)
Patient connection, insight, and clinical impact	**Connection and meaning in work** He had just experienced the loss of his partner and I got to talk to him a lot about his spiritual beliefs…. and really his comfort in the whole disease process. We also got time to focus on his home situation. He took a lot of pride in the repairs that he did in his trailer, making sure that he was able to get around. We just really focused on a lot of aspects that you don't really normally get to talk about, which was very, very nice. (R1-P6) …it was nice to be able to sit down for a couple hours and chat with someone about the human experience. Get to know somebody. I still remember my patient very well. (R1-P3) I kind of let the patient talk and tell his stories, and when you let someone share their story you end up learning a lot more and in ways that you can't really gather just from asking direct questions…. So I just really enjoyed listening to his stories and kind of learning about his personality through his storytelling…. [HTH] gave me that opportunity to [write about a patient] and remember “Okay this is nice. I like doing it and it makes me remember all the good things about medicine and patient care and the humanistic side. That's why I think it's kind of a nice thing to do intern year. You start to lose sight of that. (R1-P5) It was really nice to get to know a patient and not always be thinking about the next step for them. (R2-P2) **Insight about social context** I think he maybe enjoyed the experience of opening up more about his life and the experiences he had and how it influenced his approach to what he wanted in terms of treatment of his chronic disease and he ended up dying like two weeks after I completed this story. (R1-P5) [She] had a lot of stories about the grit of her family members, and I got a sense of what she respected, and what her values were and what makes her tick, and how she might respond to medical recommendations in the same vein….I think getting a sense of what her values were and how she saw the world allowed me to better understand how to approach her care and how to make recommendations in a way that she would understand—and how to make the right recommendations for her. (R2-P5) The thing that I got out of it the most was acknowledging how much somebody’s propensity to come to the hospital depends on their diagnosis and the stereotypes that go along with it. [My HTH was] with a person who had cirrhosis. He was a drinker, but not more than anybody that he knew…but he was really stigmatized for being an alcoholic despite having another serious liver disease [hemochromatosis] that was really the main culprit for his cirrhosis, not his drinking. His drinking just made his liver disease worse. He really had struggled with coming to the hospital because he didn’t want to be judged by nurses, doctors….(R2-P2) [HTH] helped identify some risk factors …. He had worsening dementia…[leading to] a lot of inappropriate social situations where he would say these random things out in public to people that were way off topic and his wife had a really hard time dealing with that for a number of years before even a diagnosis was made. And then he was having afib, refractory to his ablations, so he was having congestive heart failure and that was landing him in the hospital multiple times as he would forget to take his medications. (R2-P4) **Clinical impact** The major takeaway I got from him…was that he felt like his psychotherapy was not helpful for him at all...he felt…it would be better with like a group scenario or just some sort of mix and match. So wouldn't be all on him.... And so I made the recommendation when I saw he no-showed his next one-on-one psychologist's appointment. And I felt like I knew why, because he didn't think it was helping him at all. (R1-P1) From what I heard from the [inpatient] team…it was really, really helpful for their dispo[sition] planning. Because my patient was wheelchair bound, he was making a lot of modifications to his home and…it may have helped them hook him up some services to help make the improvements…sway their decision on rehab vs. home, because he seemed to have a lot of things at home that he may not have gotten at rehab. So it was really, really useful for them from that perspective. (R2-P1) I remember one of my WWII vets, a 94-year-old gentleman who had a lot of health issues, I remember reading his [HTH note] [done by another resident] and what stuck out was that he was always happy with VA care and now he just wants to be happy and comfortable, which is reasonable for someone who is 94 years old. But I think before I saw that [HTH note], I was working him up pretty aggressively for certain things…. (R2-P1)
Clinical skill development	**Social medicine learning** …that was actually one of the first times I actually had an extensive goals of care discussion with a patient, because we do a palliative care rotation as a second year, but I hadn't ever done a real conversation like that as an intern, by myself. So I felt a little bit out of my element. But I think what it helped me understand was that there were very clear ways of asking and identifying what [patients’] goals are and I think they're really closely tied to a lot of the social determinants …that was something that I think lasted with me and helped me…. (R1-P3) I think it helped me be a good PCP [Primary Care Provider]. It helped me ask the sort of questions that probably don't often get asked of [patients] when they're in contact with the health care system. So I thought that was a good learning experience.... (R1-P2) And it definitely did help become an exercise for myself not only to get to know the Vet[eran]s, but… to navigate these questions that could have been more awkward … if they've never shared these things…. It was good for me to have that conversation. (R1-P4) I am on an inpatient cardiology unit now and it makes me think a lot about how we sort of need to get on board with what the patient would like more. We do various procedures and we can run a lot of diagnostics and it’s easy to get very mechanical about it without seeing the greater objective. But what [HTH] allowed you to do, in an [inpatient] context where I can’t usually do this, is in a methodological way get a sense of who this person was and what matters to them and what the next steps will look like for them. (R2-P3) What struck me most about this project was how difficult it was to complete it…I was finally able to find the right place and time to interview my patient. And then she was so resistant to giving me details, I had to come back several times to get her full story. She would either get tired, or say you know what I just want to eat right now would you mind coming back later? And I think that highlights the importance of doing this for people, for these Vets, because oftentimes you do uncover these really important details…and it’s hard to find these really important details in the chart, in a good place, and it’s hard to get these details from them, when you are busy and they don’t want to talk, or for whatever reason. So, I think it’s great to have these really concise and loaded stories right up front in their chart to be able to access in terms of “what is their story, what is their social history, and what are the important things about them and their medical history that we should know about, that we don’t always ask about?” (R2-P5) The history is just hard and sometimes impossible to flush out with your actual patient. And a lot of us do inpatient medicine and the social aspect is ever-present but also very hard sometimes to get especially with cognitive impairment and it requires digging through the chart but also talking to family members, other people, and this was really good to learn how to really spearhead that—definitely have something really concise that everyone can go back and reference for the future—and also actually taking the time to reach out to family and understanding the context. (R2-P6) **Reflection on the physician-patient relationship** …being a good PCP I think entails— and it is really, incredibly hard— having a really good sense of what is happening with your patient in a bunch of different respects. A lot of that falls within the medical context but a lot of it also falls under…disparities and social determinants of health. So I feel like in that sense doing this type of exercise where we're kind of forced to go really, *really* farther in depth than I think I've done for any of my clinic patients …forced me to think about how much I actually know my patients. Do I really know them that well at all? I may know their medical problems but do I actually know who they are? That's what I think, not just because of this project but it did help facilitate it. Just to take a little bit more time and like get to know people. (R1-P3) Now that I am a little more efficient, I can ask more personal questions. And I think that makes a huge difference for me. I feel a lot more satisfied in the work that I’m doing just knowing the patients a little but more and feeling like a doctor who cares about somebody, versus somebody who is just trying to get through the process. (R2-P5) **Connection with other learning experiences** Morning rounds are like: “get stuff *done*” and afternoon rounds are like: “How are you? Who are you? What's going on?….it helps form alliances with the patients, it makes it more enjoyable for us. And I don’t know if I can say it's because of HTH that I do that. But it's nice and it's a similar sort of aspect. (R1-P1) …for me, this was also kind of influenced by an attending I worked with shortly after, but whenever I feel like I am struggling to get a medical history out of somebody, I skip everything and go to their social history first. Because a lot of times if you can figure out what they did, or what they use to do, or who they live with…they see that you are listening to them…and when you flip back to your more medical questions, the conversation can sometimes be easier when you know who they are as a person. And you don’t have to ask the bajillion questions of HTH…. (R2-P2)
Structural barriers to the practice of social medicine	**Systems barriers to patient expression** The patient that I interviewed … was a little bit taken aback by someone who asked the in-depth questions that we asked as a part of…an in-depth social history. I don't think anyone had ever really done that for her before….no one in the health care system had ever asked her those questions. (R1-P3) [After] explaining to him what the purpose of my interview was…he gave me a furrowed brow and then asked me “So what is this really? Are you here to sort of talk about my symptoms and management plan more, or what?” And so that was kind of awkward. (R1-P4) **Systems barriers to resident elicitation of social context** I think that something we always are limited by is the amount of time we have with people. (R1-P5) And it was a nice exercise, and I think it was refreshing in the fact that it was a time where you could just take a step back from the daily chaos, of getting things done, rounding, and notetaking, whatever it might be, and actually connect with someone, which is somewhat of an idealistic viewpoint of medicine.... But in that respect … it did remind me that: “oh yeah, there's this whole other part” after the other 70 percent that I *had* to do. (R1-P4)

Participant notation: R1-P1 denotes the first-year resident focus group in April 2019, Participant 1; R2-P3 denotes the second-year resident focus group in February 2019, Participant 3

Direct quotations from residents recorded during focus group sessions, coded by theme. Some quotations may to apply to more than one theme

#### Patient connection, insight, and clinical impact

Residents described ways in which the interview experience fostered connection and meaning in work:

He had just experienced the loss of his partner and I got to talk to him a lot about his spiritual beliefs….and really his comfort in the whole disease process. We also got time to focus on his home situation. He took a lot of pride in the repairs that he did in his trailer, making sure that he was able to get around. We just really focused on a lot of aspects that you don’t really normally get to talk about, which was very, very nice. (R1-P6)

They described examples of how these connections led to important insights about context and the social determinants of health:

getting a sense of what her values were and how she saw the world allowed me to better understand how to approach her care and how to make recommendations in a way that she would understand—and how to make the right recommendations for her. (R2-P5).

he was really stigmatized for being an alcoholic despite having another serious liver disease that was really the main culprit for his cirrhosis, not his drinking….He really had struggled with coming to the hospital because he didn’t want to be judged by nurses, doctors….(R2-P2).

[HTH] helped identify some risk factors….He had worsening dementia….[and] congestive heart failure and that was landing him in the hospital multiple times as he would forget to take his medications. (R2-P4).

Knowledge of social factors for some patients also directly impacted clinical team decision making, with implications for value-based care:

Because my patient was wheelchair bound, he was making a lot of modifications to his home and…it may have helped [the inpatient team] hook him up some services to help make the improvements…sway their decision on rehab vs. home, because he seemed to have a lot of things at home that he may not have gotten at rehab. So it was really, really useful for them from that perspective. (R2-P1).

I remember one of my WWII vets, a 94-year-old gentleman who had a lot of health issues, I remember reading his [HTH note] [done by another resident] and what stuck out was that he…just wants to be happy and comfortable, which is reasonable for someone who is 94 years old. But I think before I saw that [HTH note], I was working him up pretty aggressively for certain things…. (R2-P1).

#### Clinical skill development

Residents identified HTH as an opportunity to develop key clinical skills around complex communication and eliciting patient goals:

…that was actually one of the first times I actually had an extensive goals of care discussion with a patient….it helped me understand that there were very clear ways of asking and identifying what [patients’] goals are and I think they’re really closely tied to a lot of the social determinants …that was something that I think lasted with me and helped me…. (R1-P3).

But what [HTH] allowed you to do, in an [inpatient] context where I can’t usually do this, is in a methodological way get a sense of who this person was and what matters to them and what the next steps will look like for them. (R2-P3).

Several residents reflected the clinical importance of social history, while acknowledging the practical challenges found in working to strengthen the physician-patient relationship:

…it’s hard to find these really important details in the chart, in a good place, and it’s hard to get these details from them, when you are busy and they don’t want to talk….it’s great to have these really concise and loaded stories right up front in their chart to be able to access in terms of “what is their story, what is their social history, and what are the important things about them and their medical history that we should know about, that we don’t always ask about?” (R2-P5).

…being a good PCP I think entails— and it is really, incredibly hard— having a really good sense of what is happening with your patient in a bunch of different respects. A lot of that falls within the medical context but a lot of it also falls under…disparities and social determinants of health (R1-P3).

Other residents drew important connections to other learning experiences affecting their narrative practice:

…for me, this was also kind of influenced by an attending I worked with shortly after, but whenever I feel like I am struggling to get a medical history out of somebody, I skip everything and go to their social history first. (R2-P2).

#### Structural barriers to the practice of social medicine

Several residents identified systems barriers to patient expression of social history, such as lack of familiarity with the idea that sharing social information might have an important valence on treatment.

The patient that I interviewed … was a little bit taken aback by someone who asked the in-depth questions that we asked as a part of…an in-depth social history. I don’t think anyone had ever really done that for her before…(R1-P3).

Other residents focused on systems barriers to resident elicitation of social context in daily work.

…it was refreshing in the fact that it was a time where you could just take a step back from the daily chaos, of getting things done, rounding, and notetaking, whatever it might be, and actually connect with someone, which is somewhat of an idealistic viewpoint of medicine....(R1-P4).

## Conclusions

We developed, piloted, implemented, and evaluated qualitative learning outcomes from a social medicine consultation curriculum for first year internal medicine residents intended to advance the practice of narrative social medicine, and foster meaning in work. The opportunity to hear, co-produce, and reflect on a single in-depth patient story during the first year of residency generated educational and clinical value that was then integrated with other learning experiences reported by residents. Our data suggest that this experience brought them closer – at least transiently – to the applied practice of narrative social medicine and fostered connection with their patients.

Unsolicited, multiple residents encouraged rotating medical students to conduct similar consultations using the HTH model, signaling both a perception of value and a meaningful change in the practice of those residents. At the program level, a group of medical students designed and implemented an HTH-derivative geriatric narrative telemedicine elective in the spring of 2020, in response to concern for isolation in the face of COVID-19 [15]. We are also actively adapting this model for a new WRJVA-based IM resident telemedicine elective.

The complexity of social medicine makes it ideal for this kind of co-produced resident education, in which educators partner with learners to craft the learning experience [16]. Though co-production was not formally introduced to learners, the idea permeates the process at several stages: residents identify patients in dialogue with clinical teams; explore patient values and experiences in order to produce recommendations for care; and then return the written document to patients for review before inclusion in the EHR. In this way, residents strive to render what matters to patients, and draw on it to create shared recommendations.

HTH challenges contemporary assumptions that link burnout to time spent writing [17], which may ignore the value – to writer, reader, and patient - of what, how, and for whom a note is written. Implicitly, HTH provides an opportunity to depart from the convention of writing for the system, and to spend time writing with and for the patient in that system. Both listening and writing are powerful modes of reflection on the meaning of clinical practice. We observed that residents struggled—and perhaps also benefitted—most in writing, specifically in pivoting from the patient narrative into developing concrete recommendations. Creating these recommendations requires integrating both patient and health system knowledge; HTH demands that residents are able to locate opportunities within the patient narrative to strengthen patient-centered care in our particular clinical system. Therefore, this is not unexpected for first-year trainees, who have both limited clinical experience and limited local system knowledge within the VA, which has different patient care architecture than the more familiar academic tertiary care context in which they do most of their clinical training.

Our study has several limitations: the small size of our residency; varying intervals between the experience and its evaluation (due to immovable resident rotation schedules); the inherent difficulty of recruiting residents for voluntary focus groups due to call schedules and workload; and risk of recruitment of residents with particular interest in narrative and social medicine. These factors present challenges to generalizability, though our anthropology colleagues posited that these intimate focus groups allowed for balanced engagement and contextual richness that may have been muted in individual or larger group settings. While focus groups were intended as a mechanism of program evaluation, they may also have strengthened the educational impact and durability of the HTH curriculum through reflection, linking these principles to other learning experiences, allowing further processing and consolidation.

Our study was limited to assessment of qualitative resident learning outcomes and self-described clinical attitudes and behaviors, not measured change in observed behaviors and patient outcomes. This parallels a potential limitation of the HTH intervention itself: from a health systems perspective, there are important financial and operational barriers to more widespread implementation (many of which are captured in resident descriptions of systems barriers voiced during the focus groups). The HTH curriculum engineers space and time within the clinical learning environment for trainees to conduct these consultations, and for medically and socially vulnerable patients to share narrative in an unhurried, supportive context. In many health systems striving for clinician productivity and patient access, such an intervention may be viewed with skepticism, especially when carried out by a physician. However, given recent population health interest in interventions that meet the needs of “high needs, high cost” populations receiving frequent acute care, that skepticism may underestimate both the educational and the direct system benefit, which may defy measurement [5, 18, 19].

With these limitations in mind, HTH invites several opportunities for improvement and further study. First, deliberate integration into longitudinal curricula – such as iterative continuity clinic experiences - could offer residents practice honing the complex skill set required to carry out social medicine in the interprofessional team context with minimal operational cost [18]. Completing multiple interviews over the course of initial training would allow for accelerated connection to complex patients and to the teams required to care for them, helping advance competency in systems-based practice and practice-based learning and improvement: important, but elusive domains in medical education [19, 20]. Such integration may also have implications for resident joy and meaning in work in their continuity clinics. In our study, residents describe their unequivocal desire to deepen these formative doctor-patient relationships during their training. Second, this longitudinal structure would allow residents to observe or measure patient clinical outcomes for selected patients throughout their training. Evaluation of clinical actions initiated as a result of these consultation notes may include change in acute care use patterns [5]; instances of error mitigation (e.g. through correction of erroneous EHR data) [1]; and improved patient experience of care [2]. Furthermore, assessment of broader influences on the clinical environment, including qualitative analysis of impact on patients and interprofessional clinical teams reading these notes—and textual analysis of HTH consult note content—would give a more complete view of program value. Such analyses are needed to strengthen incentives for health system leaders to provide support for any personalized care delivery model for complex patients [5, 21]. Third, in many team-based care settings, HTH could be readily carried out collaboratively, not just by residents. Interprofessional team dialogue could further enrich the medical trainee experience of working in a system of care. The integration of social workers, case managers, nurses, subspecialists, pharmacists, community health workers, and various health professions learners would allow for multiple perspectives to identify health systems resources and opportunities, calibrate patient care goals and priorities, and foster learning across teams.

In summary, HTH adds a novel approach for teaching social medicine through the integration of narrative and health systems thinking, while bringing learners back to the bedside to find shared purpose in the stories of their patients.

## Supplementary Information

**Additional file 1.** Supplementary Digital Appendix 1: HTH Description and Curriculum Objectives for Residents. Project description and curriculum objectives sent to first-year residents by e-mail at the start of their month-long outpatient experience at the WRJVA.

**Additional file 2.** Supplementary Digital Appendix 2: HTH Social Medicine Consult Template. Simplified evidence-based social history guide for resident interviews. Residents are encouraged to include their own questions and to follow patient cues.

**Additional file 3 S**upplementary Digital Appendix 3: Structure of HTH Consultation Notes in the EHR. Screenshot of the HTH consult note, showing how the “My Story” note can be located easily from the “Postings” section of the “Coversheet” – the main dashboard of the VA EHR.

**Additional file 4.** Supplementary Digital Appendix 4: HTH Qualitative Semi-Structured Interview Guide for Resident Focus Groups. Questions used to guide discussion during resident focus groups in February and April of 2019.

**Additional file 5.** Supplementary Digital Appendix 5: HTH Qualitative Focus Group Code Descriptions. Themes derived from inductive coding of resident focus group interview transcripts.

## Data Availability

Raw qualitative datasets recorded and analyzed during the current study are available from the corresponding author on reasonable request.

## Abbreviations

HTH: Healing Through History curriculum
IM: Internal medicine
SDH: Social determinants of health
EHR: Electronic health record
GME: Graduate medical education
WRJVA: White River Junction VA Medical Center
PCP: Primary care provider
