# Supplementary material for: Healing Through History: a qualitative evaluation of a social medicine consultation curriculum for internal medicine residents
Source: BMC Med Educ. 2021 Feb 8;21:95. doi: 10.1186/s12909-021-02505-1 (PMC7869072; doi:10.1186/s12909-021-02505-1)
Supplement: Supplementary file 1 — Additional file 1. Supplementary Digital Appendix 1: HTH Description and Curriculum Objectives for Residents. Project description and curriculum objectives sent to first-year residents by e-mail at the start of their month-long outpatient experience at the WRJVA. [file 12909_2021_2505_MOESM1_ESM.docx]

**Supplementary Appendix 1: HTH Description and Curriculum Objectives for Residents**

**Healing Through History (HTH) Curriculum Objectives**

Dartmouth Hitchcock Internal Medicine Residency/White River Junction VA Medical Center

**Background**

HTH is a local effort to re-centralize the stories of medically and socially complex Veterans in the VA EHR (CPRS) through “social medicine” consultations. “Social medicine elucidates how patients’ environments influence their attitudes and behaviors and how patients’ agency— the ability to act in accordance with their free choice — is constrained by challenging social environments.^1^ The goal of these consultations is to improve understanding of Veteran social context, health experiences, clinical needs, goals, and opportunities for additional supports, taking a broad view of their life experiences and health. These HTH consultations are also an opportunity for you to sharpen your social history taking skills; take time with a patient to hear their lived experiences; and write in a way that integrates what you have learned into the plan of care, while working closely with a Veteran.

**Process**

Interviews are conducted by resident physicians, reviewed with dedicated faculty, returned to the patient for review and approval, and then published to the “Postings” section of the CPRS as a “My Story” note. This note should include 3 key components:

1. The Veteran’s “life story” as they chose to tell it.
2. A detailed social medicine interview.
3. A summary assessment, and a list of recommendations for the care team co-produced with the Veteran

Distribution to relevant clinical team members is encouraged.

**Curricular objectives**

1. Construct an approach to taking a thorough social and behavioral determinants of health history with a complex patient.
2. Develop insight about social factors that may impact clinical care, such as unmet health needs, preferences and goals of care, structural barriers, and other social determinants of health.
3. Demonstrate how co-produced patient-centered care—with attention to the doctor-patient relationship—yields important clinical insights for both patients and clinical teams.
4. Partner with a patient and interprofessional colleagues in identifying and documenting opportunities to improve care using available health system resources.

**Faculty**

Emily Cohen and Joel Bradley

**References**

1. Behforouz HL, PK Drain, and JJ Rhatigan. Rethinking the Social History. *N Engl J Med* 2014; 371; 14
2. Blumenthal D et al. Caring for High-Need, High-Cost Patients—an Urgent Priority. N Engl J Med 2016; 375: 909-911.
3. Gawande, A. Medical report: The Hot Spotters. *The New Yorker*. January 11, 2011.
