# Supplementary material for: Healing Through History: a qualitative evaluation of a social medicine consultation curriculum for internal medicine residents
Source: BMC Med Educ. 2021 Feb 8;21:95. doi: 10.1186/s12909-021-02505-1 (PMC7869072; doi:10.1186/s12909-021-02505-1)
Supplement: Supplementary file 2 — Additional file 2. Supplementary Digital Appendix 2: HTH Social Medicine Consult Template. Simplified evidence-based social history guide for resident interviews. Residents are encouraged to include their own questions and to follow patient cues. [file 12909_2021_2505_MOESM2_ESM.docx]

**Supplementary Digital Appendix 2: HTH Social Medicine Consult Template**

[Patient ID]

Interview conducted on [Date] by [Interviewer]

PART 1: My Story

[Text of story]

==============================================================================

PART 2: Social and behavioral determinants of health screening

HOUSING STATUS:

HOME LIFE:

PRIMARY LANGUAGE:

RELIGION:

LEVEL of EDUCATION:

VISION:

HEARING:

SPEECH:

COMMUNICATION PREFERENCE (PHONE, EMAIL, VA HEALTH RECORDS):

READING LEVEL:

EDUCATIONAL PREFERENCES FOR HEALTH INFORMATION:

BARRIERS TO CARE:

RELATIONSHIP WITH HEALTH CARE TEAM:

OVERALL ESTIMATE OF CURRENT HEALTH (OUT OF 10):

HEALTH GOALS:

FEARS FOR HEALTH:

STRESSORS:

ADVANCE DIRECTIVE & DPOA:

MILITARY:

WORK AND/OR WORK HISTORY:

POSSIBLE OCCUPATIONAL EXPOSURES:

HOBBIES:

MENTAL HEALTH EXPERIENCES:

SAFETY:

TOBACCO USE:

ALCOHOL AND OTHER DRUGS:

SEXUAL ACTIVITY:

LEGAL ISSUES:

==============================================================================

ASSESSMENT:

RECOMMENDATIONS:
