# Supplementary figures and images for: Healing Through History: a qualitative evaluation of a social medicine consultation curriculum for internal medicine residents
Source: BMC Med Educ. 2021 Feb 8;21:95. doi: 10.1186/s12909-021-02505-1 (PMC7869072; doi:10.1186/s12909-021-02505-1)

**Supplementary Digital Appendix 3: Structure of HTH Consultation Notes in the EHR**


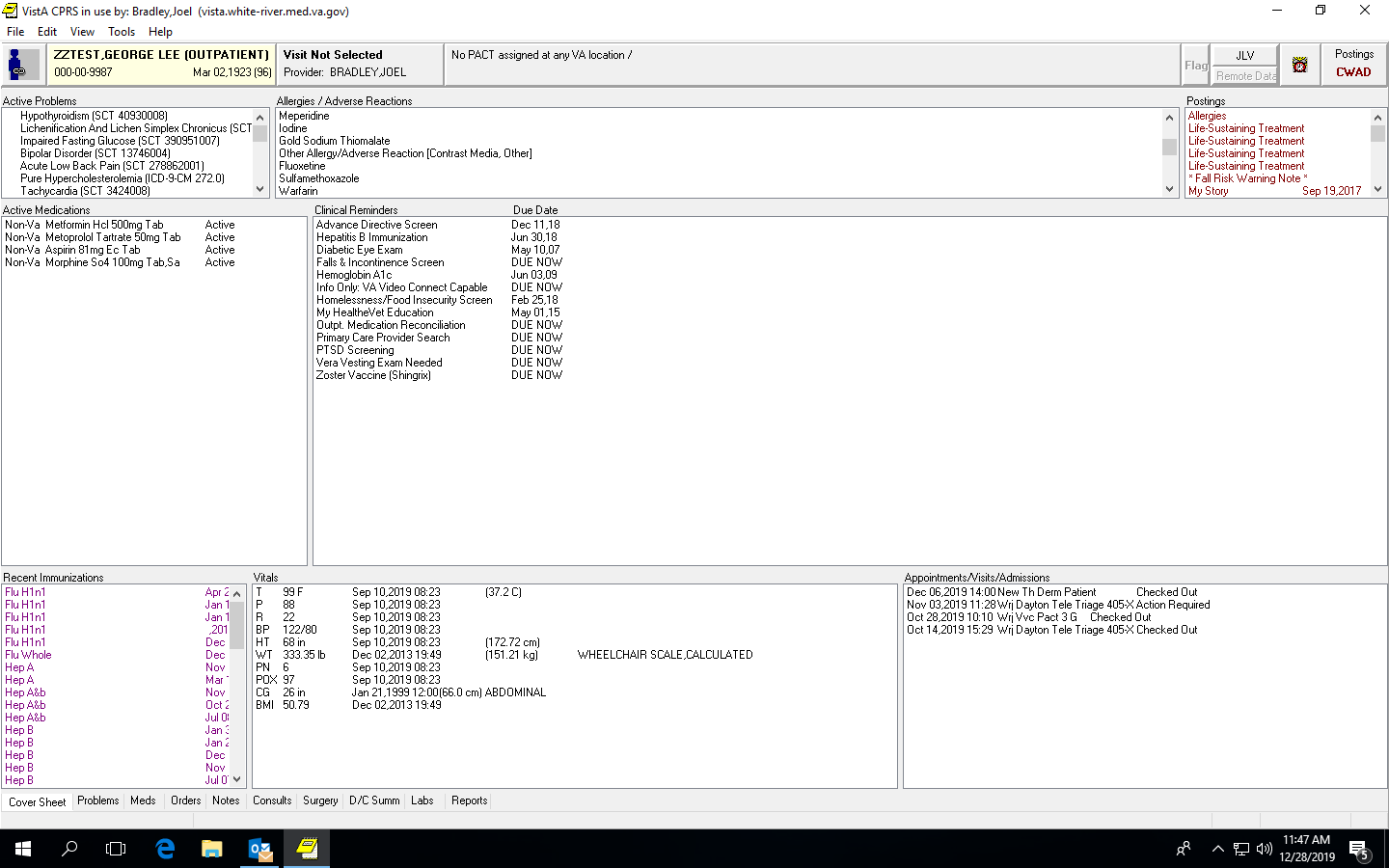


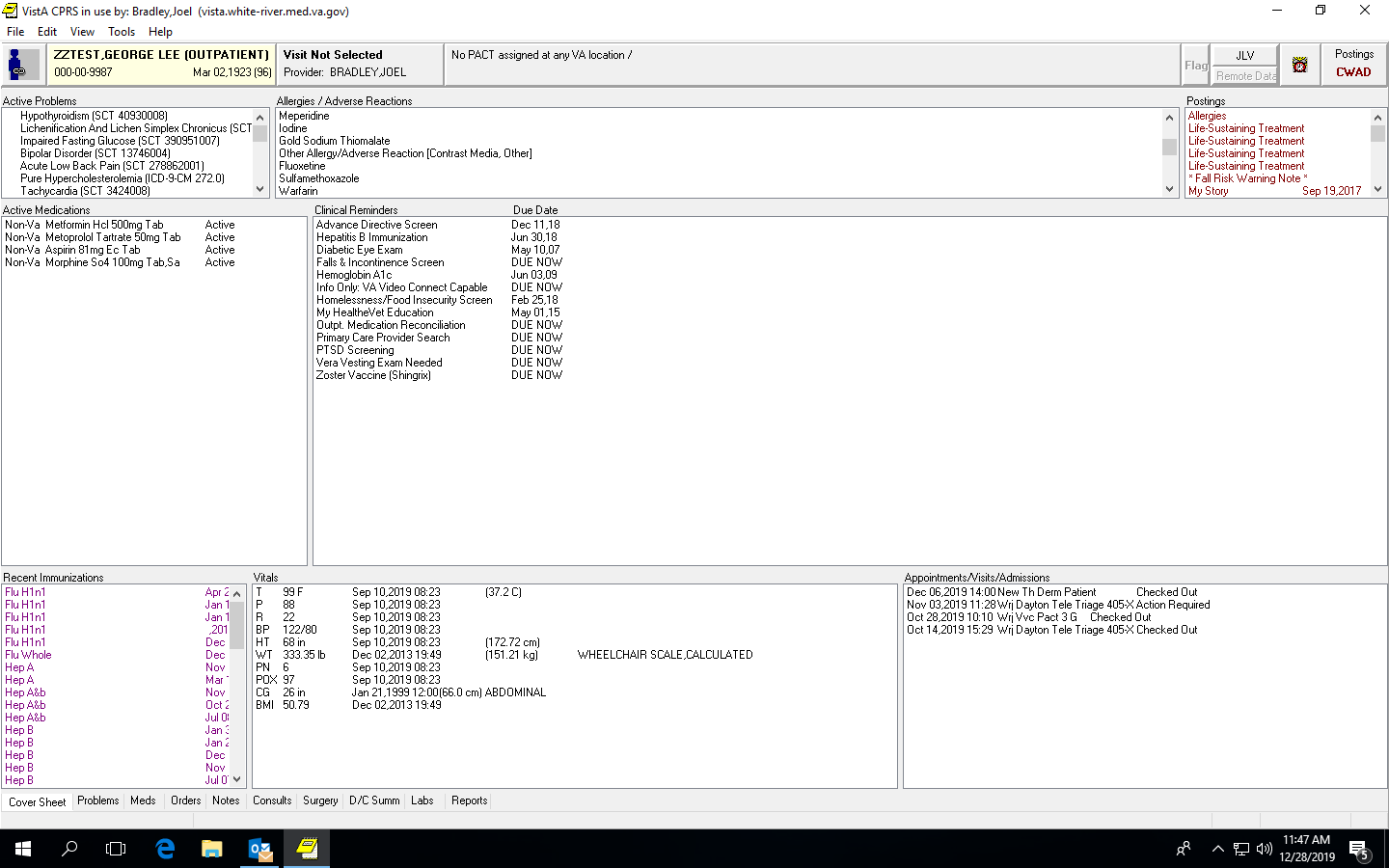


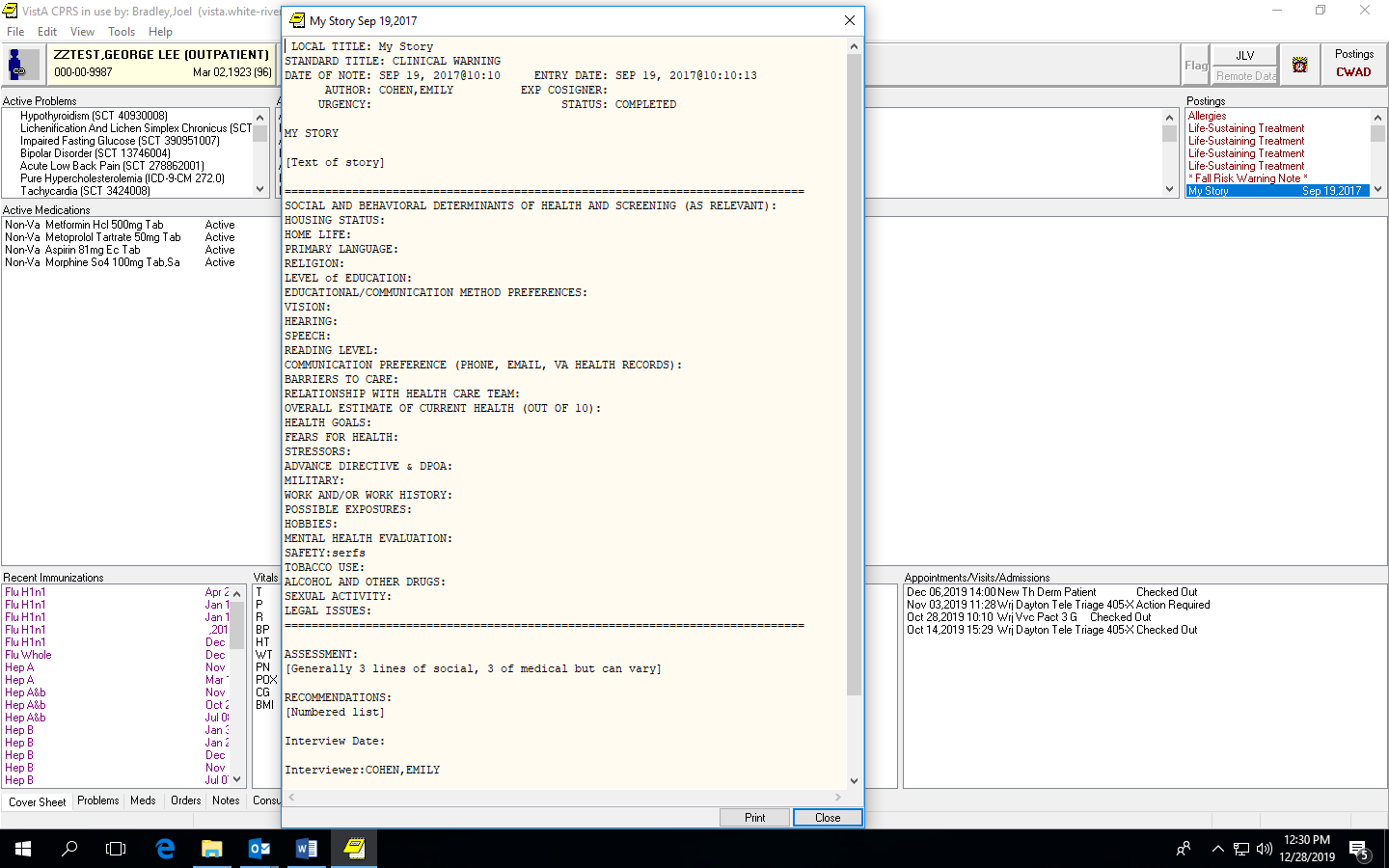

Supplement: Supplementary file 3 — Additional file 3 Supplementary Digital Appendix 3: Structure of HTH Consultation Notes in the EHR. Screenshot of the HTH consult note, showing how the “My Story” note can be located easily from the “Postings” section of the “Coversheet” – the main dashboard of the VA EHR. [file 12909_2021_2505_MOESM3_ESM.docx]
