# Supplementary material for: Healing Through History: a qualitative evaluation of a social medicine consultation curriculum for internal medicine residents
Source: BMC Med Educ. 2021 Feb 8;21:95. doi: 10.1186/s12909-021-02505-1 (PMC7869072; doi:10.1186/s12909-021-02505-1)
Supplement: Supplementary file 4 — Additional file 4. Supplementary Digital Appendix 4: HTH Qualitative Semi-Structured Interview Guide for Resident Focus Groups. Questions used to guide discussion during resident focus groups in February and April of 2019. [file 12909_2021_2505_MOESM4_ESM.docx]

**Supplementary Appendix 4: HTH Qualitative Semi-Structured Interview Guide for Resident Focus Groups**

1. Tell me about your experience with the HTH curriculum? What is one thing that struck you? One thing that you learned?
2. What do you see as the impact of the HTH curriculum? Your interaction with your patient?
3. How did gathering a detailed social history on a complex patient allow you to engage differently with the patient? How was this different than your usual work?
4. Did completing the My Story Program encourage you reflect about your own journey through medicine? How does this project relate to your original reasons for entering this profession?
5. Have you noticed a difference in how you think about work? If you were afforded time to get to know the patient at this level, do you think it would impact the meaning of your work?
