# Supplementary material for: Healing Through History: a qualitative evaluation of a social medicine consultation curriculum for internal medicine residents
Source: BMC Med Educ. 2021 Feb 8;21:95. doi: 10.1186/s12909-021-02505-1 (PMC7869072; doi:10.1186/s12909-021-02505-1)
Supplement: Supplementary file 5 — Additional file 5. Supplementary Digital Appendix 5: HTH Qualitative Focus Group Code Descriptions. Themes derived from inductive coding of resident focus group interview transcripts. [file 12909_2021_2505_MOESM5_ESM.docx]

**Supplementary Digital Appendix** 5: HTH Qualitative Focus Group Code Descriptions

| **Theme** | **Definition** | **When to use** | **When not to use** |
| --- | --- | --- | --- |
| Patient connection, insight, and clinical impact | Instances of connection with the patient leading to meaning in work; insight about elements of the social history and SDH that enhanced understanding of patient context; identification of factors directly impacting patient care, such as current health needs, preferences, and goals of care. | When the participants indicated that HTH fostered meaning or connection with the humanistic aspects of medicine; deepened understanding of social context or environmental factors affecting health; or elicited details necessitating change in the treatment plan or other clinical action. | When participants discuss patient connection, insight, or clinical impact related to practices or experiences not influenced by or relevant to HTH. |
| Clinical skill development | Description of how HTH led to a change in resident knowledge, skills, attitudes and behaviors related to: social medicine (social history interviewing, integrating social context into care plans using co-production, eliciting goals and preferences for care); reflection on the physician-patient relationship as part of clinical care; and connection with other social medicine learning experiences during clinical training. | When participants described learning methods for engaging patients, building rapport through interviewing skills and genuine inquiry, navigating difficult dynamics, obtaining a structured and detailed social history, and integrating contextual and environmental factors into the plan of care.  When residents reflect on the physician-patient relationship as part of clinical care.  When HTH connected with other resonant learning experiences. | When participants discuss clinical knowledge, skills, attitudes and behaviors that were not influenced by or perceived as connected to HTH. |
| Structural barriers to the practice of social medicine | Identification of systems factors that inhibit patient expression or resident elicitation of a patient’s social context, environment, goals and preferences of care. | When participants identified patient experiences of and expectations for health care that impacted co-production.  When participants identified structural aspects of daily work such as time, administrative burdens, or other priorities detracting from the patient-physician relationship, the practice of social medicine, or co-production. | When participants identify structural barriers on the part of patients or residents unrelated to current or prior experiences in healthcare. |
